# Assessing the Structure of Protic Ionic Liquids Based on Triethylammonium and Organic Acid Anions

**DOI:** 10.1021/acs.jpcb.1c00249

**Published:** 2021-03-10

**Authors:** Enrico Bodo, Matteo Bonomo, Alessandro Mariani

**Affiliations:** † Chemistry Department, University of Rome “La Sapienza”, Piazzale A. Moro 5, 00185 Rome, Italy; ‡ Department of Chemistry, NIS Interdepartmental Centre, INSTM Reference Centre, University of Turin, Via Gioacchino Quarello 15/A, 10125 Turin, Italy; § Helmholtz Institute Ulm (HIU), Helmholtzstrasse 11, Ulm 89081, Germany; ∥ Karlsruhe Institute of Technology (KIT), P.O. Box 3640, Karlsruhe 76021, Germany

## Abstract

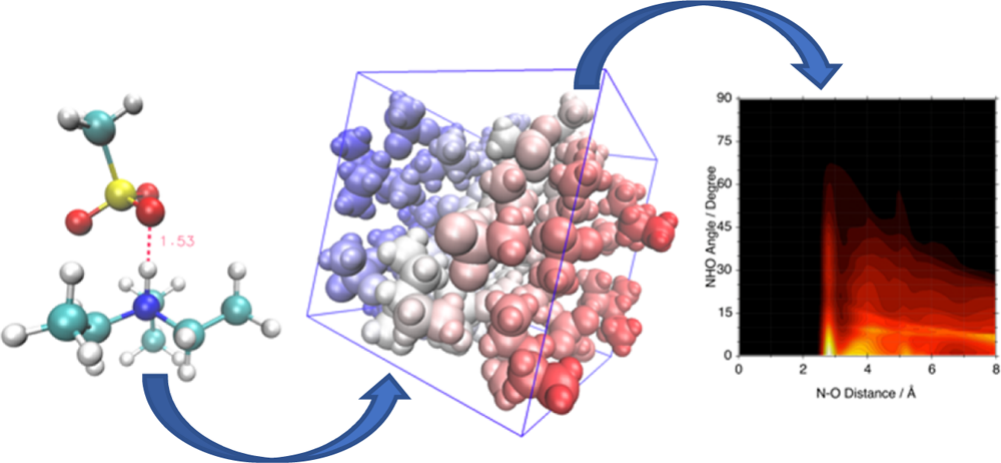

We present a computational analysis of the short-range structure of three protic ionic liquids based on strong organic acids: trifluoracetate, methanesulfonate, and triflate of triethylammonium. Accurate *ab initio* computations carried out on the gas-phase dimers show that the protonation of triethylamine is spontaneous. We have identified the anion-cation binding motif that is due to the presence of a strong hydrogen bond and to electrostatic interactions. The strength of the hydrogen bond and the magnitude of the binding energy decrease in the order trifluoroacetate ≳ methanesulfonate > triflate. The corresponding simulations of the bulk phases, obtained using a semiempirical evaluation of the interatomic forces, reveal that on short timescales, the state of the three liquids remains highly ionized and that the gas-phase cation-/anion-binding motif is preserved while no other peculiar structural features seem to emerge.

## 1 Introduction

Protic ionic liquids (PILs)^1−4^ have attracted considerable research efforts because they possess unique qualities with respect to other traditional solvents: gentle solvation property,^5^ biocompatibility,^6,7^ low toxicity,^8^ and electrochemical stability.^9^ Other attractive characteristics lie in the fact that they are synthesized using simple acid–base reactions^10−12^ and that they can be obtained from readily available and cheap ingredients such as amines and organic acids including aminoacids.

Although, in practice, a metathesis reaction with salt exchange has been proven to be a more controlled way of obtaining PILs,^13^ for all intents and purposes and for the sake of the following discussion, we shall think of them as stemming from a simple acid/base reaction:
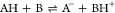
1where the proton moves from the acid onto the base, forming two molecular ions. A prototypical example of this reaction is the formation of ethylammonium nitrate which, incidentally, is one of the first ILs ever discovered.^14^

In order for reaction 1 to form a fully ionized liquid, the proton transfer from A to B must be quantitative so that all neutral components disappear. From basic chemistry, however, it is well known that reaction 1 is an equilibrium process that can be more or less shifted to the right depending on the proton donor/acceptor propensities of the two reagents involved. One of the most and readily available indicators of these propensities is the aqueous p*K*_a_. Unfortunately, the p*K*_a_ depends strongly on the dielectric properties of water and on its solvation ability toward the ionized forms in the right-hand side of reaction 1, and its application in the context of PILs is not entirely justified.^13^ Nevertheless, it has been found that a very large difference in p*K*_a_ (Δp*K*_a_) between AH and BH^+^ (greater than 10 units) generally indicates the formation of a completely ionized medium.^2,15^ In all other cases, however, the fate of reaction 1 is very difficult to predict from p*K*_a_.^16^ In these cases, it was found that the gas-phase-proton affinities of AH and of B may be alternative and more reliable indicators,^17^ although other factors such as entropy^18^ and the (self-)solvation properties of the final liquid toward its own ions^19,20^ play a crucial role as well. The net results of these considerations are that, apart from limiting cases, the factors governing the position of the equilibrium condition in reaction 1 consist of a rather complex interplay of single-molecule properties, many-body bulk phenomena, and thermodynamic conditions.^21^

A clear example of a situation where typical molecular indicators can lead to the wrong conclusion about the ionic state of a PIL is triethylammonium acetate ([TEA][Ac]). In principle, judging from Δp*K*_a_, one should expect to have an almost complete acid/base reaction and a fully ionized liquid, but it has been shown^22,23^ that [TEA][Ac] is only partially ionic with a majority of neutral components.

The presence of loosely bound protons that might be able to diffuse independently of the heavy ions (e.g., through a Grotthuss-like mechanism) is of crucial importance for the use of these compounds as electrochemical solvents.^23,24^ Hence, the determination of the proton mobility, the position of equilibrium in reaction 1, and the extent of proton binding have been the subject of previous studies.^4,24^

In this work, we present a set of calculations in order to describe the nanoscopic structure of three different PILs, all based on TEA cations coupled to anions of organic acids with increasing p*K*_a_:^25^ trifluoro acetate (TFA, p*K*_a_ = −0.25), methane sulfonate (MS, p*K*_a_ = −2.6), and triflate (Trf, p*K*_a_ = −14.7).

Pulsed field gradient nuclear magnetic resonance (PFG-NMR) was used to determine the diffusion coefficients of [TEA][Trf]^26^ at 100 °C, and it was found that the proton diffusion essentially matched that of TEA nitrogen, thereby indirectly proving the quantitative formation of the ammonium moiety. Similar evidence for a quantitative protonation of the amine in [TEA][MS] has been reported in ref (27) where, again, PGF-NMR has shown how the proton diffusion is in accord with the physical state of a liquid populated by ammonium cations. The same experiments were repeated for [TEA][TFA],^28^ and it was found that at 100 °C it can be seen as an ionic system with anions and cations diffusing together in the liquid as associated pairs. Shmukler et al.^9,29^ have presented IR vibrational spectra for several ammonium-based ILs, including the three compounds of interest here. In these works, the authors have recognized the formation of the ammonium ion vibrational bands associated with N–H motions. More recently, evidence has emerged,^30^ which partially contradicts previous determinations for [TEA][TFA] and indicates that, at room temperature, ionization may not be complete but only partial. The authors have presented PFG–NMR diffusion coefficients that, at least at room temperature, show the proton diffusing with the acid moiety.

In order to explore the behavior of these systems, we have adopted a multi-scale set of calculations. We shall use (first principles) *ab initio* calculations on isolated molecules and dimers to gather information on the pair interactions, and we shall employ molecular dynamics (MD) to explore the bulk phase. From the point of view of MD simulations, these kinds of liquid systems represent a true challenge for computational methods: on the one hand, the extent of equilibrium of reaction 1 is not known a priori and one, ideally, would like to be able to predict its fate from first principles; on the other hand, the timescales upon which equilibrium of reaction 1 is established is not known either. Due to these limitations, classical force field-based MD^31^ cannot be employed here as it generally relies on a fixed chemical topology (bonding patterns) while the position of the proton is not known. We have therefore decided to use a variant of MD where the interatomic forces are computed by differentiating the electronic energy obtained from the electronic Schrödinger equation. This kind of MD (often called *ab initio* MD or AIMD^32,33^) has the advantage that the chemical bonding scheme of the atoms involved stems directly from the electron density and is not determined a priori as in classical MD. Another advantage is that in such methods, polarization effects, charge transfer, and other many-body phenomena are naturally accounted for, without resorting to rather arbitrary charge scaling procedures.^34^ The main disadvantage is that the performance is poor, and the size of the simulated systems and the timescales of the simulation are limited. In order to improve the performance, we have used a semiempirical method (density functional tight binding, DFTB) to evaluate the electronic energy, which maintains a good accuracy at a reduced computational cost and has been tested extensively by us and others in similar systems before.^23,35−38^ Despite this, the major drawback of this approach remains the limited simulation times (∼400 ps) in which our simulations might not be able to reach a steady-state condition for equilibrium of reaction 1 especially if the systems tend to be trapped in a metastable state due to the relatively high viscosities of these substances (∼30–150 mPa s^30^) that make the dynamics of the ions extremely sluggish. In summary, our approach has several advantages over parametrized, force-field-based methods: it incorporates quite naturally many-body effects emerging from polarization of the electronic densities; it accounts for charge transfer between the ions and the related electronic quantum delocalization phenomena; it provides a full anharmonic description of the molecular vibrations, hence allowing for a quite accurate description of vibrational properties such as IR absorption spectra. It is clear that the limits of our simulations are to be found in the short times that, besides equilibration of reaction 1, are largely insufficient to reliably evaluate the frictional properties of the fluids such as viscosities or diffusion coefficients.^39^

## 2 Methods

The problem of determining the structure of these compounds, namely, [TEA][TFA], [TEA][MS], and [TEA][Trf] has been here approached using a multiscale analysis including *ab initio* computation on isolated molecules and semiempirical MD of the bulk phases.

The *ab initio* calculations have been performed using the Orca program^40^ and the MD simulations have been carried out using the DFTB + code.^41^ Analysis has been carried out with in-house codes and Travis.^42^

The *ab initio* methods used are CCSD (in its DLPNO implementation^43,44^) and four variants of DFT, namely, B3LYP,^45^ D-B3LYP (with BJ3 damping),^46^ PBEh-3c,^47^ and DSD-BLYP.^48^ All these methods, except PBEh-3c, have been used with the def2-TZVP basis set and the RI-JK approximation. The energy decomposition analysis of pair interactions has been performed using the DLPNO-CCSD method as implemented in Orca.^49^ Natural bonding orbital (NBO) analysis has been performed using the D-B3LYP orbitals.^50^

The MD simulations have been carried out with the 3*ob* parameter set^51,52^ and the third-order expansion of the DFTB energy with self-consistent charges (SCC-DFTB3).^53^ The timestep was set to 1 fs with no constraints on distances. When needed, temperature control has been achieved using the *NVT* ensemble with a Berendsen thermostat and a time constant of 10 fs.

Two kinds of simulations have been carried out. The first set of three simulations was done on small, isolated clusters of three ionic couples with no periodicity. A short, preliminary dynamic of 10 ps has been performed to thermalize the systems at 250 K, a temperature sufficiently low to avoid unwanted cluster fragmentations. Afterward, a 300 ps production run has been done in the *NVE* ensemble to collect the short-range structural feature of the systems under dynamical conditions.

Six other simulations have been carried out in a cubic box with *xyz* periodic boundary conditions. Two type of cells have been used: a small one with side of 17 Å and a large one with a side of 20 Å as detailed in Table 1. The larger cells were used only as a control test and we shall not report extensive data from them. Production times are about 400 ps (see Table 1), while the initial configurations were equilibrated and relaxed for 100 ps before production. Reasonable fully ionic initial configurations have been obtained by packing the ions so as to match the experimental densities and relaxing the structures using the MMFF force field.

**Table 1:** Parameters of MD Simulations^a^

system	side length/Å	number of pairs	density/g cm^–3^	production/ps
[TEA][MS]	20	27	1.10	30
	17	16	1.06	380
[TEA][TFA]	20	26	1.16	30
	17	16	1.16	370
[TEA][Trf]	20	24	1.25	30
	17	15	1.27	430

a The experimental densities of the three liquids are 1.12 for MS, 1.14 for TFA, and 1.27 g cm^–3^ for Trf.^30^

A preliminary validation of the DFTB method accuracy is provided here by calculating the gas-phase proton affinities (PAs) and comparing them to experimental data.^54−56^ Proton affinities have been computed using the following formulae
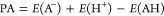
for the acids and
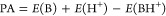
for the TEA cation, where the DFTB conventional energy of the proton has been set to 151.04 kcal/mol.^57^ The relevant data are reported in Table 2, which shows how the SCC-DTB3 method is able to reproduce quite accurately the propensity for proton capture for all compounds of this study.

**Table 2:** Proton Affinities Computed with the SCC-DFTB3 Method versus Experimental Data from Refs^54,56^

molecule	DFTB (kcal/mol)	expt. (kcal/mol)	error (%)
TEA	240.8	234.5	–2.6
TFA	332.3	323	–2.9
MS	325.3	321	–1.3
Trf	304.2	305.5	–0.4

## 3 Structure of Isolated Ionic Couples

**Figure 1:**
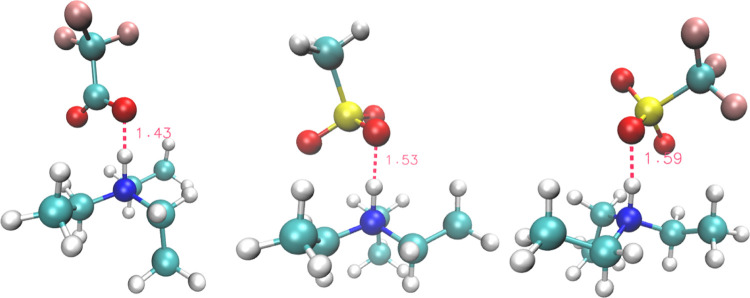
D-B3LYP/def2-TZVP-optimized structures of the ionic couples. From left to right: [TEA][TFA], [TEA][MS], and [TEA][Trf]. Proton–acceptor distances expressed in Å are reported in red.

Unconstrained D-B3LYP optimizations of the gas-phase ionic couples lead to the structures reported in Figure 1 where we have highlighted the hydrogen bonds (HBs) and the proton–acceptor distance.

Although gas-phase calculations naturally favor structures without charge separation, in all the three pairs, the migration of the proton onto the TEA molecule seems to be spontaneous, in accord with what one can predict from the values of the PAs. The molecular geometry optimizations have been repeated with the plain B3LYP, PBEh-3c, and the DFTB methods. The resulting geometric parameters relevant to the HB are reported in Table 3 for all methods plus some data gathered from literature.^58−60^ Both B3LYP and D-B3LYP essentially provide the same geometric parameters for the HB whose geometry does not seem to depend on the inclusion of dispersive interactions. DFTB, despite its semiempirical nature, provides HB geometries which are in perfect agreement with those resulting from both hybrid functionals.

**Table 3:** Geometric Parameters of the HB in the Isolated Ionic Couples, as Obtained by Different Methods^a^

molecule	method	O–H/Å	O–N/Å	O–H–N/deg
[TEA][TFA]	D3-B3LYP	1.44	2.56	178.5
	B3LYP	1.44	2.57	179.1
	PBEh-3c^b^	1.05	2.60	179.5
	DFTB	1.44	2.59	175.7
			2.56^c^	175.4^c^
[TEA][MS]	D3-B3LYP	1.53	2.61	176.4
	B3LYP	1.53	2.62	177.9
	PBEh-3c	1.43	2.54	175.7
	DFTB	1.57	2.66	176.2
		1.52^d^	2.60^d^	174.5^d^
[TEA][Trf]	D3-B3LYP	1.58	2.64	174.6
	B3LYP	1.60	2.67	174.4
	PBEh-3c	1.50	2.58	173.5
	DFTB	1.64	2.71	174.4
		1.59^d^	2.65^d^	170.2^d^

a Data from previous calculations have also been added.

b The proton is on the oxygen.

c B3LYP-GD3/6–31++G(d,p) from ref (58).

d B3LYP-GD3/6–31++G(d,p) from ref (59).

Significantly different geometries have been obtained using the PBEh-3c method which employs a “double-zeta” basis set but incorporates a larger amount of “exact” exchange than B3LYP. The greatest difference is found for the [TEA][TFA] ionic couple where PBEh-3c converges toward a neutral structure with the proton on the oxygen of the acid. We have attributed this result to the relatively small basis set employed by that method, which has no polarization function on hydrogen atoms. In order to substantiate this conclusion, we have repeated the optimization of [TEA][TFA] using the M06-2X functional which is rich in “exact” exchange: the final geometry agrees with the D-B3LYP one.

**Figure 2:**
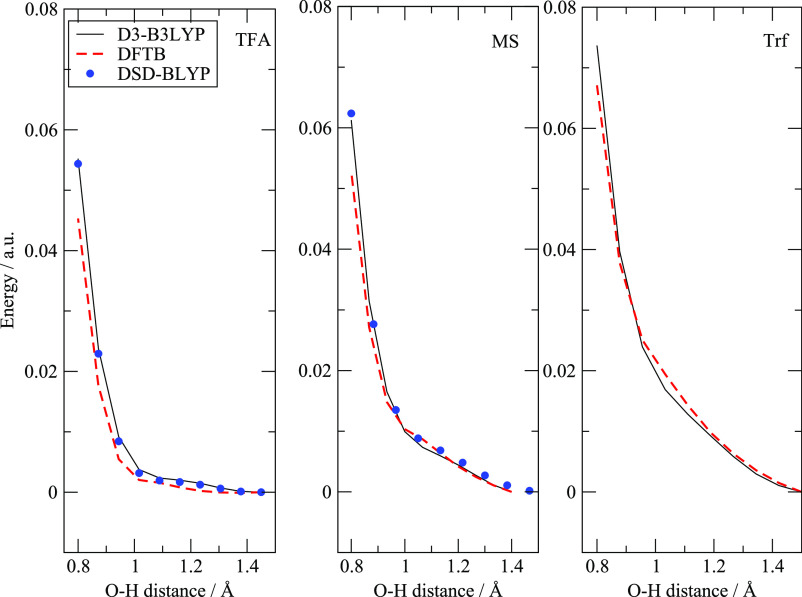
Geometric (relaxed) scan of the O–H distance in the three ionic couples: left, [TEA][TFA]; center, [TEA][MS]; and right, [TEA][Trf]. At 0.8 Å, we find a situation with the proton on the oxygen atom. At 1.5 Å, the proton is on the nitrogen. The zero has been arbitrarily set to the lowest energy values for each method.

In order to further verify that the stable state of the isolated ion couples was the one with the proton localized on the nitrogen atom a set of geometry optimization (scans) of the pairs have been performed where the O—H distance has been constrained and varied gradually from 0.8 to 1.5 Å. The results are reported in Figure 2 as potential energy curves along the O—H distance. The set of constrained geometries optimization has been calculated using the D-B3LYP and DFTB methods with the addition (where necessary) of the DSD-BLYP double hybrid functional which includes MP2 correlation and is one of the best performing functional in terms of thermodynamic accuracy.^60^ Depending on the acidity of the anionic partner, the potential energy profile, when going toward a protonated acid, is very repulsive for [Trf] and [MS], while it turns out to be less so for the least acidic [TFA]. Despite these differences, the ionic conformer with the proton on the ammine is clearly the only stable minimum of all the pairs. The energy profiles of Figure 2 are similar to those reported in refs. (4,58,59) which essentially confirm our findings except minor differences due to geometric setup and methods. Although these results cannot be used as an incontrovertible evidence of an ionized state of the liquids (due to the ionic couple being isolated), they nevertheless suggest that a complete (or at least significant for TFA) acid deprotonation in the bulk phase is very likely to occur for these liquids. The strength of this conclusion also stems from the consideration that the possible introduction of a polarizable screening media to model solvation (e.g., the introduction of a polarizable continuum model) would only increase the stability of the ionized state with respect to the neutral one.

Returning to the data in Table 3, we see how the acceptor–donor distance (N–O) increases slightly in the order [TFA] < [MS] < [Trf], while the collinearity of the acceptor–proton–donor angle decreases accordingly. Arguably, the HB strength decreases in the order [TFA] > [MS] > [Trf], which is inverse to the (p*K*_a_-based) acidity of the organic acid.

The adiabatic binding energies of the ionic couples with respect to their relaxed ionic dissociation are reported in Table 4, where we have also added the zero-point-energy (ZPE) corrected interaction as coming from a frequency calculation at the D-B3LYP level and the interaction energies coming from a single point evaluation with CCSD at the D-B3LYP geometry. The trend in interaction energies is consistent across the different methods and shows that the binding strength decreases in the order [TFA] ≳ [MS] > [Trf], which is in line with the trend in HB strengths detected above. It interesting to note how the binding energies are only slightly affected by the inclusion of ZPE corrections and how good is the performance of the semiempirical method DFTB when compared to CCSD.

**Table 4:** Binding Energies in kcal/mol of the Ionic Couples with Respect to the Relaxed Ionic Fragments^a^

	D-B3LYP	B3LYP	PBEh-3c	DFTB	D-B3LYP + ZPE	CCSD	previous calc.
[TEA][TFA]	–110.71	–105.01	–116.41	–106.70	–110.65	–109.59	–112.4^b^
[TEA][MS]	–111.39	–103.97	–114.69	–105.59	–111.02	–108.36	–113.5^c^
[TEA][Trf]	–100.17	–92.91	–102.81	–94.32	–99.53	–97.62	–101.1^c^

a Data from the literature have also been added.

b B3LYP-GD3/6–31++G(d,p) from ref (58).

c B3LYP-GD3/6–31++G(d,p) from ref (59).

By looking at the components of the vertical (geometry fixed) binding energies obtained at the CCSD level and reported in Table 5, we see that the electrostatic energy is by far the greatest contribution to the ionic couple binding energy, while exchange and dispersive components play only a minor role. The latter result agrees with the fact that the geometries computed at the B3LYP and D-B3LYP levels are very similar. The amount of charge transfer between the anion and cation (evaluated using the natural atomic orbitals populations) decreases on increasing the acidity in the order [TFA] > [MS] > [Trf]. In other words, the charge density on the anion tends to become more and more compact when its acidity increases and its propensity to remain bound to the proton decreases.

**Table 5:** Separate Components of the Vertical Binding Energy in kcal/mol, as Computed using DLPNO-CCSD at the Gometric Minimum Obtained with D-B3LYP^a^

	Δ*E*(el)	Δ*E*(ex)	Δ*E*(disp)	net charge of ions
[TEA][TFA]	–251.8	–21.2	–8.0	±0.82
[TEA][MS]	–219.0	–18.0	–8.5	±0.86
[TEA][Trf]	–182.7	–15.0	–8.2	±0.89

a The relaxation energy due to the geometric rearrangement is not included. In the last column, we report the net charges of the two ions (from natural atomic orbital populations).

**Figure 3:**
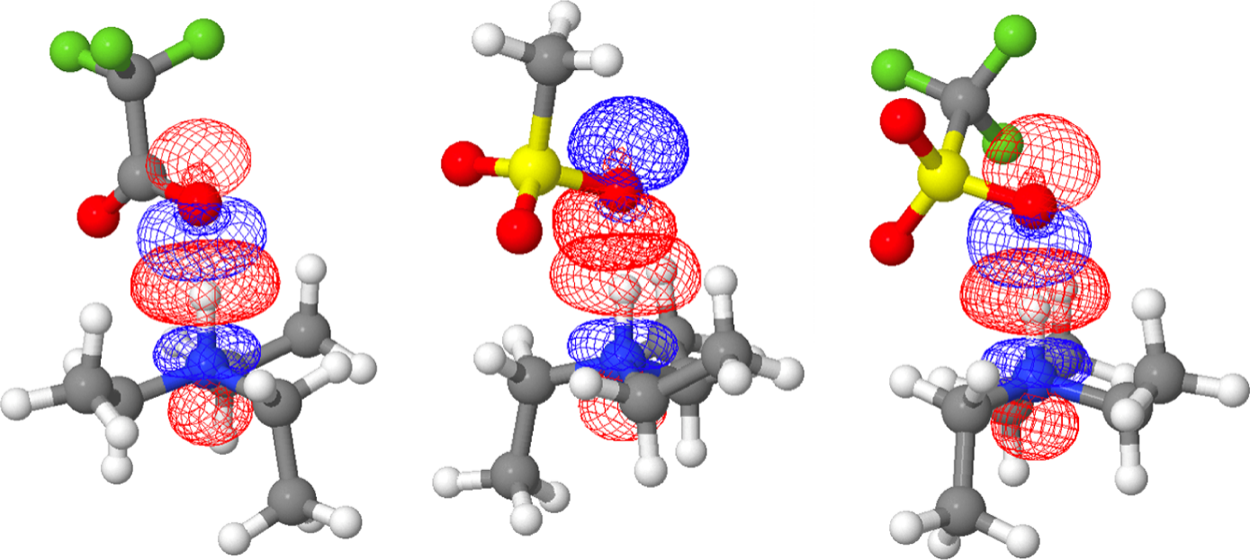
NBOs participating in the HB in the ionic couples. The first one is an atomic orbital localized on the donor oxygen (two lobes, *p*-shaped). The second one is an empty anti-bonding orbital localized on the acceptor N–H bond (three lobes, σ*-shaped). These plots contain two independent NBOs; hence, their relative phase (colors) is irrelevant. From left to right: [TEA][TFA], [TEA][MS], and [TEA][Trf].

A significant portion of the charge transfer is due to the HB, which stems, as expected, from the interaction between the doubly occupied, nearly atomic orbitals of the oxygen atoms (the lone-pairs) and the antibonding valence orbital localized on the [R_3_N–H]^+^ bond. The involved NBOs are shown in Figure 3.

**Figure 4:**
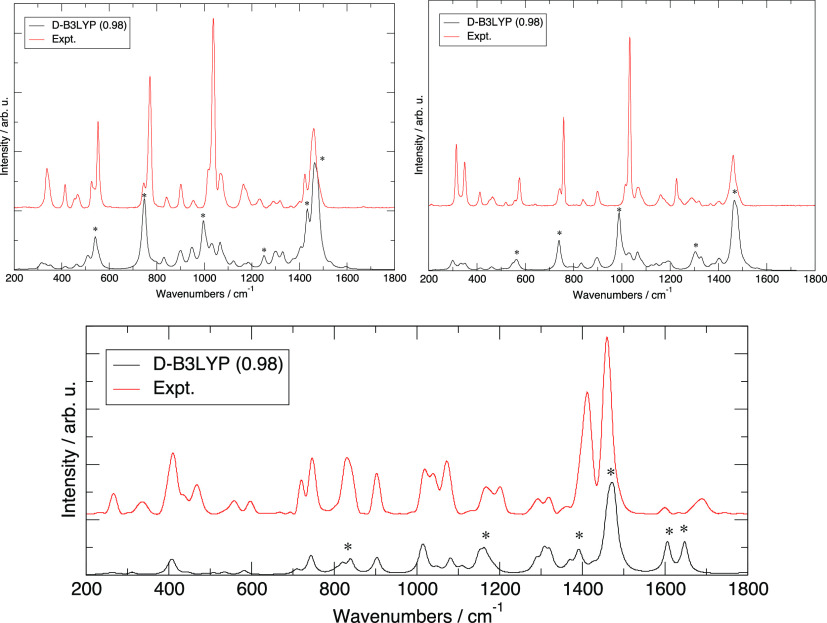
Raman spectra computed at the D-B3LYP/Def2-TZVP level compared with experimental measurements from ref (30). Left, [TEA][MS]; right, [TEA][Trf]; and bottom, [TEA][TFA]. The computed frequencies have been scaled by a uniform factor of 0.98 to account for anharmonicity.

The possibility that the general binding patterns detected in the isolated ionic couples can characterize the fluid state of the liquids can be further investigated by comparing the computed harmonic Raman spectra with the experimental ones reported in ref (30). Since the high-frequency motions of the N–H groups in the real liquids are subjected to a wide range of perturbations due to HBs within the bulk, the spectral bands are generally very broadened and the isolated ionic couple approach is inapplicable for those vibrational modes. Hence, we limit this analysis to the fingerprint region where the deficiency of the isolated couple approach and the inaccuracy of the harmonic frequencies are mitigated. The calculated and measured Raman spectra are presented in Figure 4.

Overall, the simple ionic couple model is able to account for almost all the resonances seen in the experimental Raman spectra with a very good accuracy in the position of the various bands but a certain mismatch in intensities.

The two spectra of [TEA][MS] and [TEA][Trf] show similar features. The intense peaks between 1400 and 1500 cm^–1^ are mostly due to TEA CH_*n*_ motions, including scissoring of the CH_3_ and CH_2_ groups. The SO_3_ asymmetric stretching falls at 1250 cm^–1^ in MS while turns out to be barely visible in Tr where it is coupled to the CF_3_ deformations and split into several bands, one at 1300 and another just above 1100 cm^–1^. SO_3_ symmetric stretching gives rise to the intense peaks at 995 and 990 cm^–1^ for MS and Trf, respectively, which are both slightly blue-shifted with respect to the experimental band (this depends upon the uniform scaling parameter). The C–S stretching falls at 750 cm^–1^ for [MS] and 740 cm^–1^ for Trf. The group of peaks at 540 and 560 cm^–1^ for MS and Trf are due to umbrella motions of the C–SO_3_ group and other deformations of the same group.

The Raman spectra for the [TEA][TFA] ionic couple are also presented in Figure 4. Few interesting differences with respect to previous spectra can be seen. At 1600 and 1650 cm^–1^, blue-shifted with respect to the peak due to TEA CH_*n*_ motions at 1470 cm^–1^, we see two distinct bands that loosely match with two experimental resonances. In the calculation, these are both due to an N–H bending motion coupled with asymmetric and symmetric stretching of the CO_2_^–^ group. At lower frequencies, the resonance at 1390 cm^–1^ is due to a C–C stretching on TFA. The peak at 1150 cm^–1^ is due to several oscillations which are coupled to CF_3_ motions. The band at 820 cm^–1^ is the only one at lower frequencies, which arises from the anion and specifically arises from a collective motion including C–C stretching and CO_2_ symmetric stretching.

## 4 Cluster MD

The modeling of the fluid state for these systems, in order to account for possible proton transfer equilibria, must not rely on the use of techniques which imply a fixed topology of the chemical structures. In addition, and since the bulk phase is dominated by local structures due to HBs, we also need a scheme able to account for charge transfer between the ions (see Table 5) and that includes polarization effects. Therefore, one way to approach reliably the study of such systems is represented by methods that evaluate the atomic forces using an approximate solution of the electronic Schrödinger equation. Such methods suffer from two main drawbacks: the simulations are very computationally demanding and their sizes and timescales are limited. A valuable approach to improve the sampling time is that of using a non-excessively expensive way to solve for the electronic energy and its gradient. Our choice is the semiempirical method based on DFTB.

Before extending the computation toward the bulk phase, we have analyzed the behavior of small clusters of ions made by three ionic couples.

**Figure 5:**
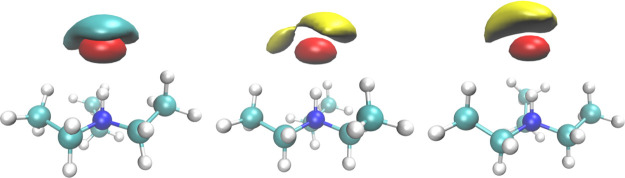
Averaged atomic densities over 100 ps MD runs for the acceptor oxygen atoms (red) and the atoms directly bound to them (cyan for C on TFA and yellow for S). From left to right: TFA, MS, and Trf.

We have found that the docking motif between TEA and the deprotonated acid in the clusters resembles closely the one noted for isolated ionic couples and that the HB remains a directional and strongly binding feature between the ions. In Figure 5, we show the average spatial density (SD) of the acceptor oxygen atoms and of the atoms directly bound to them (C for TFA and S for MS and Trf) calculated by keeping fixed in space the N–H bond. It is evident that the HB, during the cluster dynamical evolution, maintains a high directionality and strongly localizes the acceptor atom positions. It is also apparent that the presence of other surrounding ions only slightly perturbs such binding motifs.

**Figure 6:**
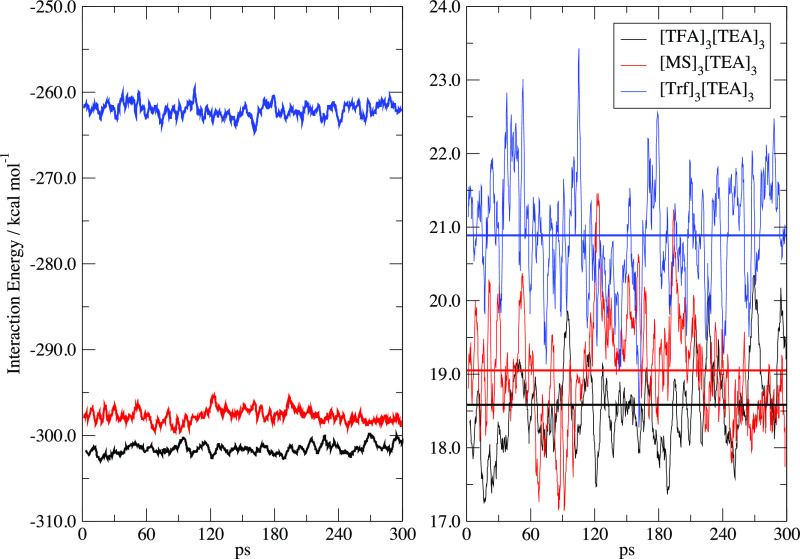
Average interaction energies Δ*E*_fi_ (left) and Δ*E*_ic_ (right) as a function of simulation time. In the right panel, to ease the visualization, the average values of the energies are shown as horizontal lines. See text for details.

The energy of the clusters along the 300 ps of simulation is shown in Figure 6 where we have extracted and reported the averaged MD potential energy (i.e., the electronic energy) and compared it with two different reference points. The panel on the left contains the binding energy of the cluster (Δ*E*_fi_) as calculated with respect to the full ionic fragmentation at the same temperature, that is, [A]_3_[TEA]_3_ → 3[A]^−^ + 3[TEA]^+^, while the right panel reports the evaporation energy (Δ*E*_ic_) as computed with respect to the fragmentation of the cluster into three separate ionic couples [A]_3_[TEA]_3_ → 3[A][TEA]. The Δ*E*_fi_ represents the total binding energy of the cluster at 250 K with respect to the fragmentation in its molecular components at the same temperature. It is a strongly negative quantity whose magnitude is slightly less than three times the binding energy of a single ionic couple. This decrease is due to the presence, in the aggregate, of repulsive like-charge interactions and to the polarization of the surrounding molecular ions that induce a general weakening of the Coulomb interactions. The least stable cluster is [Trf]_3_[TEA]_3_ in accord with its isolated ionic couple being the weakest (Table 4).

Another way of looking at the same situation is represented by the Δ*E*_ic_ in the right panel of Figure 6 where we have computed the 250 K average electronic energy in the cluster with respect to the average energy of three separated ionic couples at the same temperature. In this case, the energy of the cluster is only weakly negative and, when compared to the data on the left, shows (at least in principle) a propensity of the cluster to evaporate into neutral ionic couples instead of isolated ions. The data are substantially the same for the three compounds with a lesser tendency to evaporate for Trf than for the other two acids.

**Figure 7:**
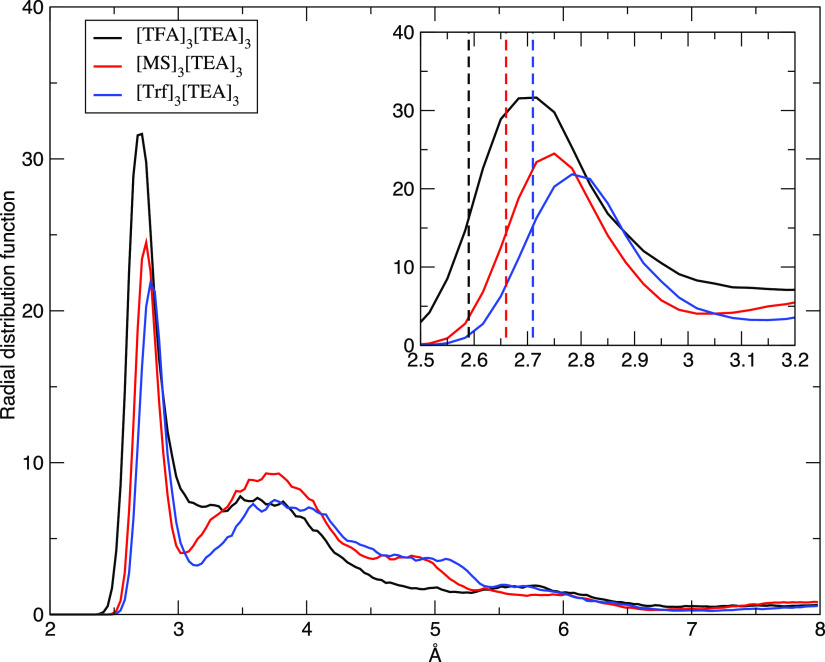
RDFs for the N–O distances in the three clusters. The inset shows an enlargement of the maximum region plus the equilibrium values of the same distances, as obtained in the isolated ionic couples.

The weakening of the binding energy per ionic pair in the cluster can be understood if we look at the radial distribution functions (RDFs) for the O–N distance reported in Figure 7 and extracted from the cluster dynamics. The average N–O distances in the HBs (the first peak in the RDFs) are consistent with the data in Tables 3 and 4 and reflect the already known trend in HB strength: [TFA] > [MS] > [Trf]. In all clusters, however, we see a sizable increase of the average N–O distance with respect to the equilibrium value obtained by isolated ionic couple optimization (Table 3). The inset in Figure 7 shows that this increase of the average N–O distance with respect to the ionic couple ideal values (vertical lines) is about 0.1 Å. Given that the HB in the ionic couple is strong, a small change in the acceptor–donor distance when increasing the system size produces a corresponding weakening of the binding energy, hence the data in Figure 6. In other words, the presence of a surrounding medium (albeit in the limited form of a small cluster) produces a slight destabilization of the ionic couples.

## 5 Bulk MD

In this section, we present the extension of the above calculations to the bulk phase via a set of DFTB-based MD simulations at 300 K using periodic boundary conditions applied to cubic cells with 17 and 20 Å side lengths (see Table 1). The initial configurations of these cells contained only ionic species.

**Figure 8:**
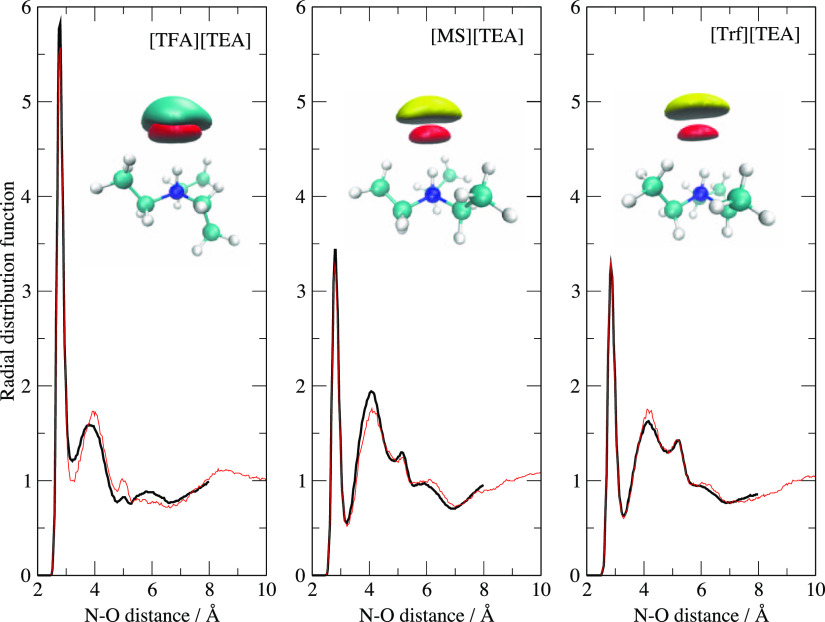
RDFs for the N–O distances in the three liquid bulk simulations. Black lines: 17^3^ Å^3^ cells. Red lines: 20^3^ Å^3^ cells. The superimposed structures depict the SDs computed in the bulk phase in the same fashion as in Figure 5.

First of all, the MD simulations show that the ionic couple structure described in the previous sections is preserved also in the liquid bulk phase. In order to explore the features of the HB, we present in Figure 8 the RDFs of the N–O distances and the SDs of the acceptor atoms. Their shapes are similar to those reported in Figure 7 since, obviously, the HB between the nitrogen and oxygen atoms is a local phenomenon only slightly altered by the presence of the surrounding bulk. The only minor change is a slight increase of the average N–O distance due to the rise in the number of surrounding molecules and therefore to their increased screening effect. The first peak in the RDF of Figure 8 is due to the HB between N and O, whereas the second and third ones (when present) are due to the second and third oxygen atoms, which are part of the same acidic functional group. The RDFs of Trf and MS are very similar in shape because they share the same SO_3_ group which, in turn, induces the same binding pattern to the N–H group. Apart from this secondary structure in a short range, no further structures are clearly detectable, thereby showing that the liquid is substantially unstructured, apart from the obvious cation/anion alternation pattern due to electrostatic interactions and from this the local docking pattern of the two counterions. No other functional group shows a particular propensity toward aggregation as can be concluded by inspection of the relevant RDFs between them (not reported).

**Figure 9:**
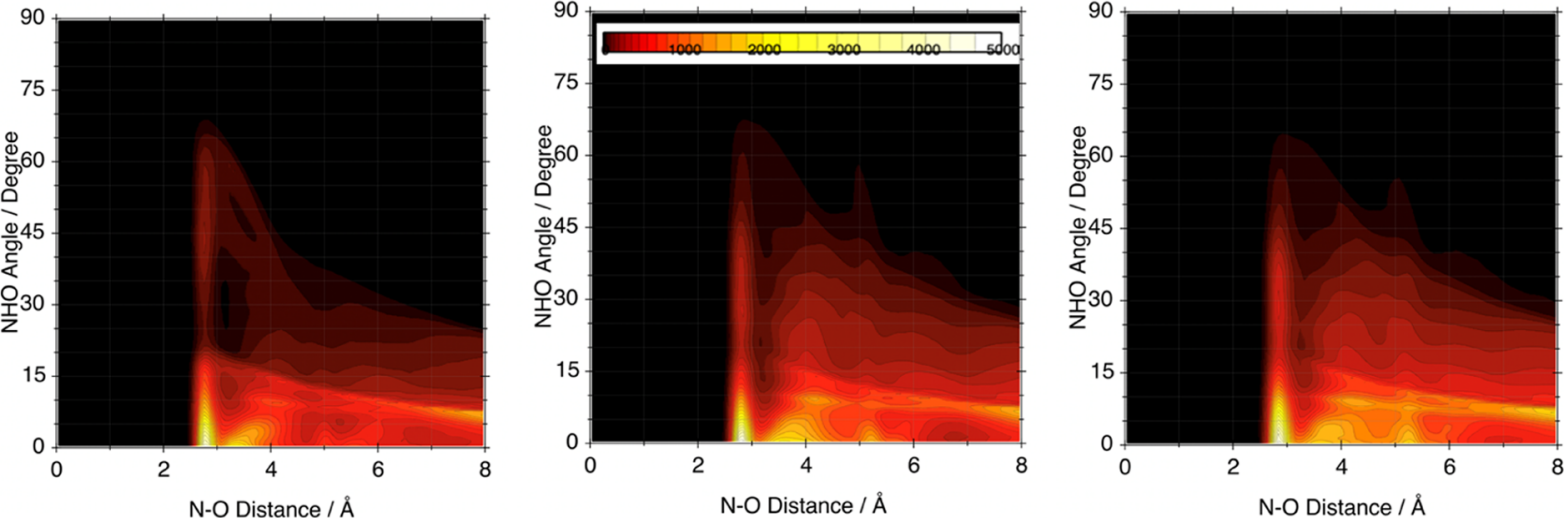
NHO angle/NO distance correlation map. Brighter regions correspond to the highest occurrence counts. Left: [TFA]. Center: [MS]. Right: [Trf].

We present a further characterization of the HB in the fluid phase of these compounds by means of the correlation between the N–O distance involved and the deviation from collinearity (the NHO angle). The results for the three simulations are presented in Figure 9 through a color map where the “hotter” colors represent maximum correlation and the dark region its absence. If we focus on the first peak at 2.7–2.8 Å, we see that the HB keeps its strong directionality also in the fluid phase with little or no correlation at angles greater than 10°. The faint extension of the peak distribution in the angular coordinate (vertical scale) is due to the transient and sporadic appearance of a double coordination of the N–H group with a second oxygen atom of the same anion. If we now move to the secondary peaks in the RDFs localized between 3 and 6 Å, we note that the angular distribution of these radial contacts is broader due to the flexibility of the docking pattern between anions and cations and the rotation of the SO_3_ and CO_2_ groups around the N–H–O axis of the HB.

One of the most common problems when dealing with ionic liquids is represented by the extremely sluggish motion of the ions in the fluid. This makes it particularly difficult to obtain reliable estimates of several dynamic quantities of interest such as viscosities, diffusion coefficients, and so on. In order to achieve a reliable computational representation of dynamical events (such as ionic diffusive motion), the simulation times should well extend beyond the ns regime. Unfortunately, this is extremely time-consuming also using the present semiempirical approach. Therefore, our investigation of the fluid dynamics is very limited and only qualitative at best. We summarize our findings in the following.

First, in our simulations, we have not detected any event of proton transfer from the nitrogen atom to the oxygen one. In other words, in our simulation we do not see the occurrence of mutual neutralization reactions. This is in line with the gas phase data we have presented in Figure 2. The formation of a neutral pair of molecules, for these acids, is energetically impossible in the isolated ionic couples. The problem with the bulk fluid is that the energetic landscape of proton transfer in the ionic couple can be substantially altered by the presence of a surrounding medium and the emergence of other stable structures cannot be excluded. The results obtained here for the MS and Trf liquids, where a fully ionized state of the liquid is preserved during the dynamics, agrees with what has been found in our recent paper.^30^ Our current computational findings however do not corroborate the evidence presented therein that the TFA liquid might not be a fully ionic system. This can be due to various reasons. First and foremost, due to the short timescales sampled here, we cannot be sure that the fully ionized state we have from our simulations is a truthful representation of the fluid thermodynamic state. In other words, although the present data tend to lean in favor of a fully ionized state for all systems, we cannot exclude that long timescale proton exchange reactions (∼ns) could take place and alter the ionized to neutral proportion [i.e., the position of equilibrium of reaction 1]. For example, the presence of clusters of neutral species might induce a more efficient stabilization of the ions and keep ionization at a minimum, but the simultaneous formation of neutral species is an extremely unlikely event in our computational setup on both the temporal and spatial scales sampled by us. Second, the methods employed here are not “exact” but are based on a semiempirical evaluation of the electronic energy and could fail to grasp all the energetic details of the liquid environment and to determine the correct position of equilibrium in reaction 1.

As mentioned before, the dynamical quantities are difficult to converge on such short timescales, but we have at least attempted to characterize a few of them. The lifetimes of the hydrogen-bonded ionic couples can be estimated by using the continuous autocorrelation function of a simple time-dependent, binary signal which equals 1 upon the occurrence of a given geometric condition, such as the occurrence of a certain distance between the HB acceptor and donor atoms. We have computed such functions using the condition that the distance between the N donor and the O acceptor must be less than the first minimum in the RDFs of Figure 8. The lifetimes of the ionic couples turned out to be 26, 9, and 6 ps, respectively for [TEA][TFA], [TEA][MS], and [TEA][Trf], which is in line with the stability trend detected in Section 3. The events that contribute to the breaking of the ionic couples that we are able to sample are processes in which the HB is broken and reformed by changes among the three or two donor oxygen atoms and, only sporadically, processes that involve a change between two acceptor molecules. In other words, we are mostly looking at the short timescale dynamics of the HB inside a given ionic couple. We stress that the above lifetimes are not reliable measures of the stability of the bimolecular aggregates because they do not account for the fact that an HB can be reformed inside the same ionic couple. Nonetheless, they show how the typical lifetimes of HBs in the TFA liquid are much longer than in the other two, thereby implying that the dynamics of the ionic component of the TFA bulk liquid might be interpreted in terms of very stable ionic couples, while that of the other two liquids involves a greater presence of dissociated ions. For example, the TFA liquid has the lowest ionic conductivity of the three despite also having the lowest viscosity^30^ and this could be explained by the presence of neutral components and the persistence of the ionic couple aggregates in the ionic phase. In other words, the propensity for TFA and TEA to interact through very strong HBs and to exist as pairs hinders the ionic drift while, at the same time, decreasing the friction. On the other hand, the bulk properties of MS and Trf seem to adhere to the Walden rule where the viscosity and conductivity are inversely proportional.^30^

## 6 Mid-IR Spectra

The DFTB method produces fluctuating atomic charges from the atomic electronic populations. In principle, it is possible to calculate the IR absorption profiles using the autocorrelation function of the temporal fluctuation of the dipole moment of the simulated system. In practice, however, the charges derived from atomic populations are not extremely reliable and the calculated spectra often present only a qualitative agreement with experiments, especially for the mid-IR range where we find the X–H absorption. A better way to interpret the IR spectra is to resort to the so-called “power spectra”, which substantially consist of calculating the vibrational density of states (VDOS) as it stems from recurring atomic motions which are more reliable than charges. The VDOS is obtained from the Fourier transform of the velocity autocorrelation function and indicates the preferential frequencies where IR absorption can occur depending on the magnitude of the dipole variation.

**Figure 10:**
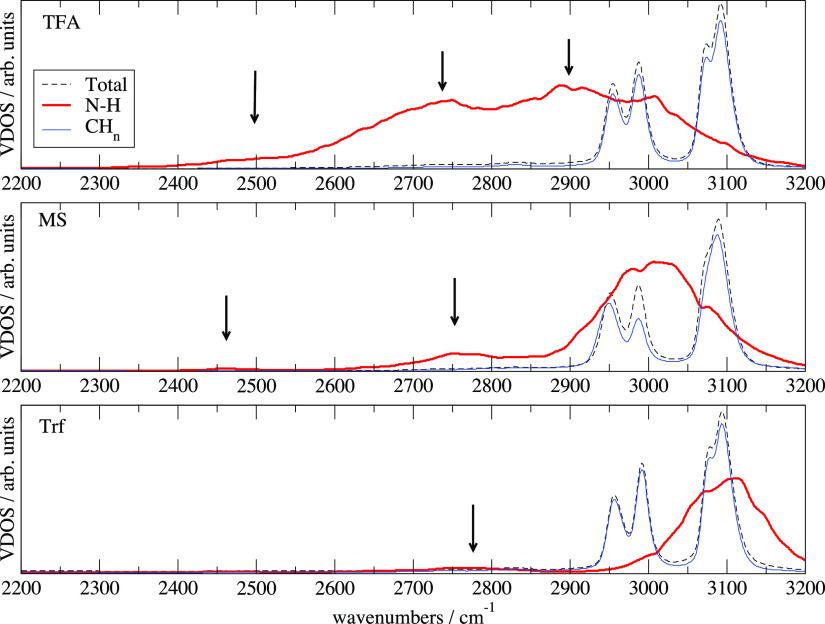
VDOS for the three liquids in the mid-IR range. In the middle panel, the contribution of CH_*n*_ includes both TEA and MS. The N–H contribution (in red) has been enlarged to make it visible on this scale. The arrows indicate less visible features of the VDOS because of the scale choice.

The reference experimental IR data can be found in ref (30) (specifically in Figure 3). The computed VDOS for the three liquids are reported in Figure 10 in the range 2200–3200 cm^–1^. Therein, we show the total VDOS of the system and its components due to the N–H and the CH_*n*_ groups of the of TEA cation. The arrows in Figure 10 indicate the presence of features in the VDOS that are not clearly visible on the chosen scale but when coupled to a sizable dipole moment variation might induce a substantial IR absorption.

The three sharp VDOS features between 2900 and 3200 cm^–1^ are due to the stretching motions of the TEA CH_*n*_ groups and are common to all the three liquids. The VDOS produced by the motion of the N–H bond is much broader and unstructured because of the tight HB network in which it is involved. The position and width of this absorption simply reflect the strength of the HB between anions and cations (hence, also the lifetime of the ionic couples mentioned above). In TFA, the HB in the ionic couple is the strongest and the motion of N–H is strongly perturbed by it giving rise to the broad band in the range 2400–3100 cm^–1^. When moving to the weaker HB in MS, the vibration of N–H is more localized in the 2900–3100 cm^–1^ range although we can trace residual VDOS features at 2450 and 2750 cm^–1^. In the case of Trf, the N–H VDOS appears clearly blue-shifted and the only surviving feature at low frequencies is located at 2800 cm^–1^. These results are in qualitative agreement with the data from ref (30), apart from a uniform adjustment/scaling of the frequencies, which is necessary to correct for the systematic errors linked to the classical treatment of the nuclear motion.

## 7 Conclusions

We have reported a new set of calculations on three PILs: [TEA][TFA], [TEA][MS], and [TEA][Trf]. Our analysis is based on accurate *ab initio* calculations of the isolated pairs and semiempirical MD simulations of small clusters and bulk fluids. The *ab initio* calculations reveal that the stable state of the isolated ionic pair is one where the two molecules are ionized with the proton on the nitrogen atom of the amine. The binding pattern of the isolated ionic pair is determined by a strong HB between the donor nitrogen of the amine and the acceptor oxygen atom of the acid. The trend in stability of these pairs is bound to the stability of this HB and the ionic pair binding energy turns out to decrease in the order [TFA] ≳ [MS] > [Trf].

The structure of the hydrogen-bonded ionic pair is quite rigid with very directional HB with an almost collinear arrangement of the acceptor–H^+^–donor complex. This binding motif survives with only a very minor alteration when we expand the size of the system by including more than one ionic pair in a small cluster. A general decrease of the overall binding energy has been noted in the clusters and attributed to the appearance of like-charge repulsion and to the polarization effect due to the surrounding molecules that weakens the electrostatic interactions.

MD simulation of the liquids tells us that the anion–cation binding motifs detected with *ab initio* are preserved in the bulk phase of these liquids. The HB features substantially maintain the same structure found in the isolate ionic pairs with the same trend in the stability reported above. From the point of view of dynamics, an analysis of the aggregation conditions in the bulk fluid reveals that, among the three fluids, the [TEA][TFA] ionic pairs have the longest lifetime in accord with the computed binding energies. This preferential pair aggregation in [TFA] might explain partially some of its bulk properties such as a peculiar low ionic conductivity.

In all the simulations, evidence of mutual neutralization reactions did not emerge within nearly 400 ps of evolution time. Nevertheless, we cannot exclude these reactions to emerge on longer timescales (e.g., ns). In this case, for example, the possible formation of highly stable hydrogen-bonded dimers of neutral species (i.e., bidentate trifluoroacetic acid dimers) could drive the system toward a lower ionization degree. In other words, with the methodology employed here, we have been unable to corroborate the interpretation recently reported in ref (30) where several experimental data have been presented in support of a sizable neutralization of the [TEA][TFA] liquid. This discrepancy, if confirmed, clearly highlights how even seemingly simple systems such as those examined here can give rise to a surprisingly complex chemistry that represents a true challenge for current computational methods.
